# Establishing the China‐SCO Cooperation Center for Metabolic Diseases to Build a Prevention and Control System for Metabolic Disorders, Advancing the Global Community of Shared Future

**DOI:** 10.1111/1753-0407.70211

**Published:** 2026-04-19

**Authors:** Guang Ning

**Affiliations:** ^1^ Ruijin Hospital, Shanghai Jiao Tong University School of Medicine Shanghai China

To tackle the significant health challenges posed by metabolic diseases, the China‐SCO (Shanghai Cooperation Organization) Cooperation Center for Metabolic Diseases was officially launched at Ruijin Hospital, Shanghai Jiao Tong University School of Medicine on February 27, 2026. The center will integrate the strengths of member states in both traditional medicine and modern medical technologies, promote standardized diagnosis and treatment protocols, and establish a coordinated regional prevention and treatment network. National Metabolic Management Centers (MMC) have already been implemented in 2068 medical institutions nationwide in China, managing over 3.5 million patients—a successful model for regional collaboration. The center will focus on disease surveillance, technological innovation, talent development, and experience sharing, aiming to build an equitable and inclusive healthcare service system. It will provide strong support for improving the health of 3.4 billion people in the SCO region while contributing regional expertise to global metabolic disease prevention and control efforts

Regional collaborative prevention and treatment of metabolic diseases is a core issue concerning the health and well‐being of the 3.4 billion people within the SCO. On November 18, 2025, at the 24th Meeting of the Council of Heads of Government (Prime Ministers) of the SCO Member States in Moscow, Premier Li Qiang solemnly proposed “the establishment of a China‐SCO Cooperation Center for Metabolic Diseases, aiming to improve the well‐being of the people in all member states.” This proposal is both a concrete practice of the global governance initiative—“promoting the establishment of a more just and equitable global governance system and jointly advancing a community with a shared future for mankind”—put forward by President Xi Jinping at the SCO Tianjin Summit on September 1, 2025, and aligns with the overarching vision of “building a global community of health for all” championed by President Xi Jinping. It represents a “SCO demonstration of promoting shared human values” and holds landmark significance.

Metabolic diseases—a term that may sound highly professional, yet it concerns the health of every individual. These conditions include not only diabetes, obesity, gout, and cardiovascular diseases but are also closely linked to cancers and serve as a major risk factor for accelerating aging. They represent one of the most serious health challenges facing humanity in the 21st century.

The SCO faces an even more severe threat from metabolic diseases. The diabetes prevalence rate within the SCO far exceeds the global average, and the growth rate is even faster. Metabolic diseases and their associated complications—such as cardiovascular and cerebrovascular diseases, renal failure, blindness, and amputations—have become the leading cause of mortality from noncommunicable diseases within the SCO, accounting for over 70% of all deaths. Significant differences exist among member states in genetic backgrounds, cultural customs, dietary structures, economic levels, and disease susceptibility. How can consensus be sought amidst such national diversity to establish a fair, equitable, inclusive, and universally beneficial prevention, control, and governance system for metabolic diseases? This requires multilateral coordination mechanisms, clear top‐level design, comprehensive institutional policies, effective resource integration, and equitable, universally accessible safeguards. The global health governance system for conditions such as metabolic diseases is a vital component of President Xi Jinping's “initiative for a just and equitable global governance system” and is closely tied to the enhancement of people's well‐being.

It is encouraging that SCO countries have accumulated extensive local experience in the prevention, control, and governance of metabolic diseases. In China, Russia, Egypt, Turkey, and Central Asia, long‐standing civilizations have also nurtured unparalleled traditional medical systems. In China, the chronic disease management strategy that equally emphasizes traditional Chinese and Western medicine has yielded significant results. In India, yoga and Ayurvedic medicine have been incorporated into the national chronic disease management plan. In Kazakhstan and Egypt, mobile screening, remote monitoring, and mobile medical vehicles have effectively addressed the prevention and control challenges posed by vast territories and sparse populations. These successful experiences demonstrate that within the resource‐limited and culturally diverse SCO region, it is entirely possible to forge a path of “just, equitable, inclusive, and universally beneficial” prevention, control, and governance, thereby setting an “SCO model” for global metabolic disease prevention and management.

This is precisely the mission of the China‐SCO Cooperation Center for Metabolic Diseases. It is to promote regional coordination, cooperation, and experience sharing; to drive technological iteration, upgrading, and widespread adoption; to foster innovation and sustainable development in governance mechanisms; and to advance the globalization of the “SCO model.”

As a representative of the China‐SCO Cooperation Center for Metabolic Diseases, I am honored to share with you the practical experience from China. In 2016, Ruijin Hospital, affiliated with the Shanghai Jiao Tong University School of Medicine, initiated the National Metabolic Management Center (MMC) project. Guided by the philosophy of “one center, one‐stop service, one standard,” the project established 492 Standard Operating Procedures (SOPs), applied 72 core technologies—including metabolic multi‐function machines, noninvasive glucose meters, fully automated eye disease screening devices, and the “Doctor‐Nurse–Patient” app—and created a comprehensive, standardized operational and quality control system. At an MMC, a metabolic multi‐function machine can complete a comprehensive examination, diagnosis, and management plan for diabetes and its complications within 15 min. MMC's “1 + X” tiered diagnosis and treatment network enables the standardized protocols developed by top‐tier experts to be extended downward to rural and grassroots communities, ensuring that every patient, regardless of location, can access homogeneous, high‐quality medical services.

A robust data platform powered by the internet, the Internet of Things, and the “Chrocare” chronic disease management large language model enables seamless integration of in‐hospital and daily monitoring throughout the entire disease course, with self‐iterative capabilities. Today, this standardized health network has expanded to cover all 32 provincial‐level regions in China, including Macao. A total of 2068 hospitals have joined the MMC initiative, collectively managing over 3.5 million diabetes patients, making it the world's largest diabetes management system. By reducing hospitalization rates and complication risks among diabetes patients, MMC is projected to save more than 2 billion yuan in medical costs for society annually, significantly lowering disability‐adjusted life years (DALYs), and substantially improving the quality of life for patients.

The focus extends beyond diabetes. In 2024, China launched the National Key Research and Development Program targeting the “Four Major Chronic Diseases,” treating cancer, cardiovascular and cerebrovascular diseases, respiratory diseases, and metabolic diseases as an integrated whole to explore unified prevention, control, and governance. The National Health Commission, in collaboration with 16 other ministries and commissions, initiated a 3‐year action plan for weight management. This strategic shift in China's chronic disease control, moving from a “treatment‐focused” to a “prevention‐focused” governance system, is a vivid practice of President Xi Jinping's important health governance philosophy that “there is no comprehensive well‐being without universal health.” It also provides a replicable and scalable “China Model” for SCO countries and global health governance.

SCO Secretary‐General Yermekbayev stated at the SCO Health Ministers' Meeting in April 2025: “Only by strengthening cooperation and building a resilient, inclusive, and universally beneficial healthcare system can we jointly address the challenges of our time.”

The vision of the China‐SCO Cooperation Center for Metabolic Diseases is to forge consensus among SCO nations on metabolic disease prevention, control, and governance, and to build a “just, equitable, inclusive, and universally beneficial” SCO Metabolic Health Community that meets the well‐being needs of the people in the SCO region. To achieve this, we need to establish a regionally coordinated monitoring and early warning network and map a dynamic regional “Metabolic Disease Atlas.” We need to share experiences in metabolic disease prevention, control, and governance to form a distinct SCO‐characteristic metabolic disease prevention, control, and governance system. We need to establish metabolic disease training bases to cultivate high‐quality personnel for metabolic disease prevention, control, and governance. We need to establish well‐equipped, technologically advanced research and development bases to develop effective technologies for metabolic disease prevention, control, and governance.

The China‐SCO Cooperation Center for Metabolic Diseases is committed to becoming a multi‐level, networked, and intelligent regional health cooperation center. It aims to build an international medical services hub within the region; a dual‐track precision talent training base within the region; a strategic research base for diseases within the region; and a base for cooperation in medicine and technology within the region. We will establish an expert committee for the China‐SCO Cooperation Center for Metabolic Diseases, bringing together metabolic disease experts from SCO member states to plan for the Center's development and formulate metabolic disease prevention, control, and governance strategies. We propose initiating the “SCO Forum on Metabolic Disease Governance” to share experiences, construct an SCO metabolic disease prevention, control, and governance system adaptable to each country's characteristics while possessing common features, formulate diagnosis, treatment, and intervention plans suitable for SCO population characteristics, promote the standardized MMC model, leverage artificial intelligence to realize remote and mobile healthcare, and create “Mobile Metabolic Centers.”

Presiding over the 25th SCO Summit, President Xi Jinping remarked with a profound historical perspective: “From the banks of the Huangpu River to the shores of the Haihe River, the SCO has traversed an extraordinary journey of development.” Today, on the occasion of the 25th anniversary of the SCO's establishment, adhering to the “Shanghai Spirit,” the establishment of the China‐SCO Cooperation Center for Metabolic Diseases in Shanghai carries the health aspirations of 3.4 billion people, the mission of our times to build a just, equitable, inclusive, and universally beneficial healthcare governance system, and the responsibility of “sharing SCO experience globally.” Let us join hands and forge ahead to build an SCO Metabolic Health Community, contributing SCO strength to enhancing the well‐being of all member states' people and advancing the building of a global community of health for all.

## Funding

The author has nothing to report.

## Conflicts of Interest

The author declares no conflicts of interest.

